# Impact of loop diuretic dosage in a population of patients with acute heart failure: a retrospective analysis

**DOI:** 10.3389/fcvm.2023.1267042

**Published:** 2023-11-23

**Authors:** P. Meani, M. Pagnoni, G. M. Mondellini, S. Fiorenza, H. P. Brunner-La Rocca

**Affiliations:** ^1^ Cardio-Thoracic Surgery Department, Heart & Vascular Centre, Maastricht University Medical Centre, Maastricht (MUMC), Maastricht, Netherlands; ^2^ Division of Cardiology, Lausanne University Hospital, Lausanne, Switzerland; ^3^ Division of Cardiology, Università Degli Studi di Milano, Milan, Italy; ^4^ Division of Cardiology, Istituto Clinico Città Studi, Milan, Italy; ^5^ Cardiovascular Research Institute Maastricht (CARIM), Maastricht University Medical Centre (MUMC+), Maastricht, Netherlands

**Keywords:** loop diuretics, acute heart failure, acute heart failure treatment, acute heart failure outcomes, worsening renal function

## Abstract

**Background:**

Loop diuretics are essential for managing congestion in acute heart failure (AHF) patients, but concerns exist about their dosing and administration. This study aims to explore the relationship between aggressive diuretic treatment and clinical outcomes in AHF patients.

**Methods:**

We randomly selected 370 AHF patients from admissions at Maastricht University Medical Center between January 2011 and March 2017. Patients were divided into four quartiles based on diuretic doses administrated during index hospitalization. The primary endpoint was a composite of cardiovascular (CV) rehospitalization or death at 1 year.

**Results:**

42.4% of patients experimented the primary outcome The composite endpoint rates were 35.4%, 41.6%, 38.5%, and 54.9%, respectively, from lowest to highest dose quartiles (*p* = 0.033). In univariate analysis, the outcome was significantly lower in the first three quartiles as compared to the fourth quartile. One-year CV mortality was 9.1%, 10.1%, 20.9% and 27.2%, respectively (*p* = 0.002). After adjusting for confounders, the association between loop diuretic dosage disappeared for both the primary outcome and one-year CV mortality. Most secondary outcomes and endpoints at 3 months, including worsening renal function, showed no significant differences between groups, while hypokaliemia occurrence, length of hospital stay and weight loss at index admission were higher in the fourth quartile compared to the first one.

**Conclusions:**

High loop diuretic doses are associated with poor outcomes in AHF patients, reflecting disease severity rather than harm from aggressive diuretic use. Furthermore, high diuretic doses do not seem to negatively affect kidney function.

## Introduction

Acute heart failure (AHF) is one of the most important causes of hospitalization in Western countries. High readmission and mortality rates continue to be a burden on healthcare systems worldwide (1, 2). The main reason for AHF is worsening congestion, defined as signs and symptoms of extracellular fluid accumulation that result in increased cardiac filling pressures (3).

The goal of therapy in these patients is the relief of congestion achieving a state of euvolemia, mainly through diuretic therapy. Loop diuretics, used in over 90% of patients, are the cornerstone in the treatment of congestion to reduce left ventricular filling, avoid pulmonary oedema and alleviate peripheral fluid retention (1). Despite their importance in the treatment of AHF and the ubiquity of their administration, significant concerns have been raised regarding the balance of risks and benefits, especially regarding the dosage and the administration regimen of loop diuretics.

The analysis by ADHERE Registry showed that patients receiving lower doses have a lower risk of in-hospital mortality, ICU stay, prolonged hospitalization or adverse renal effects (4). However, the DOSE trial, the largest prospective randomized double-blind controlled study assessing loop diuretics in HF, demonstrated that high dose in comparison to low dose (equal to home dose) resulted in no effect on the co-primary endpoints of global assessment of symptoms or change in serum creatinine (s-Cr) over 72 h and on 60-day death or rehospitalization. Still, it demonstrated a favourable effect on secondary endpoints of dyspnoea relief, change in weight and net fluid loss (5). In addition, the high-dose group was associated with better 60-day outcomes when adjusted for cumulative loop diuretics dose received (6).

The association between high doses of loop diuretic and worsening renal function (WRF) is of particular interest since the latter is correlated in observational studies with worse outcomes (including longer length of stay, hospital readmission and increased long term CV mortality) in the setting of AHF (7). However, in the DOSE Trial, WRF occurred more often in the high-dose group, but a *post hoc* analysis illustrated that this increase in creatinine did not portend a worse outcome (8). In this regard, transient WRF might mirror a more aggressive administration of loop diuretics with effective decongestion and be acceptable in acute decongestion, while persistent WRF is more likely due to more advanced hemodynamic impairment, kidney damage, and more intense neurohormonal activation (9). However, the relationship between decongestion, transient or persistent WRF and diuretic management is still incompletely understood, particularly in unselected AHF populations.

The primary goal of the present study is therefore to investigate the relationship between more aggressive diuretic treatment and clinical and renal outcomes in a cohort of patients with AHF of various aetiologies.

## Methods

### Subjects

In total, 3,206 patients admitted to the Cardiology department or Intensive Care Unit of the Maastricht University Medical Center (MUMC) between January 2011 and March 2017 were screened, and patients were selected using the following inclusion criteria: (1) age >18years; (2) primary diagnosis with AHF or co-primary diagnosis with newly developed HF during the index admission; (3) length of hospital stay of at least 3 days. In patients with repeated admission, only the first admission was considered. Patients with left and/or right ventricular assistant device and those were undergoing heart or heart-lung transplantation during the index hospitalisation and during follow-up were excluded from the analysis. Patients were also excluded if their medical record could not be accessed for administrative reasons. Of the remaining 2,144 patients, 370 admitted with a diagnosis of acute decompensation HF, hospitalized for at least 3 days, were randomly selected, and enrolled in this retrospective study. The number of included patients was based on a power calculation for an internal quality assessment to investigate the impact of a change in treatment regimens in ADHF (data on file), which was also the reason why the inclusion period ranged over 7 years. The clinical evaluation and AHF evaluation were performed by trained physicians when the patients were admitted at our Institution (details in Supplementary Data).

During hospitalization, every patient underwent standard clinical evaluation and received recommended heart failure treatment. The choice of diuretic regimen was left at a physician's discretion.

We collected patients' characteristics, including their age, gender, comorbidities, previous therapies, the presence of *de novo* or recurrent HF, the aetiology of HF, the risk factors for atherosclerosis, vital signs, admission symptoms and physical findings, the left ventricular ejection fraction (LVEF) on echocardiography, laboratory data, chest x-ray findings (signs of lung congestion), medications administered during the first week of hospitalization and the length of hospital stay. Obesity was defined as a BMI >30 kg/m^2^, anaemia was defined as Hb <7.5 mmol/L in women and <8.1 mmol/L in men. Heart valve disease (HVD) was considered significant in case of at least moderate degree severity.

This study complies with the Declaration of Helsinki (10) and the research protocol was approved by the local appointed ethics committee.

### Assessment of diuretics doses and quartiles definition

As loop diuretics, either furosemide or bumetanide was used. The doses of loop diuretics (oral or intravenous) during the first week of hospitalization were assessed day by day expressed as mg/24 h in furosemide equivalents. For intravenous diuretics, we considered either boluses or continuous infusion. Bumetanide doses were converted to furosemide equivalents using previously published conversions where 1 mg of bumetanide was equivalent to 40 and 80 mg furosemide for intravenous and oral administration, respectively (11). We then divided the population in 4 groups according to quartiles of daily mean dose of furosemide equivalents.

### Assessment of renal function

Serum creatinine levels, collected daily during the hospitalization from admission to day 7, were analysed. Glomerular filtration was estimated with the *Cockcroft-Gault equation*, and chronic kidney disease (CKD) was defined as estimated glomerular filtration rate (eGRF) lower than 60 ml/min/1.73 m^2^.

We set the definition of WRF during the acute phase of HF in the present study based on the evidence that almost all WRF occurs within seven days (12). WRF was defined as the occurrence during the first week of hospitalization of a ≥0.3 mg/dl (26.5 µmol/L) increase in s-Cr from admission.

### Outcomes

The primary outcome was the combined endpoint of cardiovascular (CV) mortality or CV rehospitalization over a one-year follow-up period. Secondary outcomes were three-month CV mortality or CV rehospitalization, three-month and one-year CV mortality, one-year CV rehospitalization, in-hospital CV mortality, WRF, dyspnoea relief, hypokalaemia (serum potassium <3.5 mmol/L), hyperkalaemia (serum potassium >5 mmol/L), weight loss and length of hospital stay.

### Statistical analysis

Results are presented as mean ± standard deviation (SD) for continuous variables or as median (interquartile range) for continuous variables with a skewed distribution and as percentages of the total for categorical variables. Continuous variables were compared by two-tailed paired t-test and analysis of variance in case of normal distribution, and using the Mann–Whitney *U*-test and Kruskal–Wallis *H*-test in case of abnormal distribution. Categorical variables were tested using Chi-squared tests. A *p*-value < 0.05 was considered to be statistically significant.

The treatment groups defined by dose were compared with logistic regression for binary end points. We divided our sample into quartiles according to the mean dose of furosemide equivalents. The highest quartile was used as the reference when calculating odds ratios (ORs), hazard ratios (HRs) and confidence intervals.

The association between diuretic dose with primary and secondary outcomes were tested using univariate logistic regression models. A multivariate logistic regression model was also tested for primary endpoint and 1 year CV mortality. The variables included in the multivariate model were: age, gender, obesity, diabetes mellitus, smoking, hypertension, arrhythmia, chronic heart failure (CHF), admission systolic blood pressure (SBP) ≤90 mmHg, admission creatinine, urea, sodium and N-terminal proB-type natriuretic peptide (NT-proBNP).

In order to test the association between the treatment groups and continuous variables with a linear regression model, every diuretic dose standard deviation parameter was associated with dummy variables for quartile 1, 2 and 3, hence, the unstandardized B coefficient (with confidence intervals at 95%) represented the difference from the highest quartile.

Kaplan–Meier method was used to compare those groups for time-to-event endpoints and differences between the curves were evaluated with the log-rank statistic. In addition, univariable and multivariable Cox proportional hazard models regressed time to primary endpoint on quartiles of diuretic dose, age, gender, obesity, admission systolic blood pressure (SBP) ≤90 mmHg, presence of anemia, admission sodium, creatinine, urea and NT-proBNP. BM SPSS Statistics for Windows, Version 26.0 (Armonk, NY, IBM Corp) was used for all analyses.

## Results

### Characteristics of the cohort at admission

At admission, characteristics of the study population are presented in Table 1. The age of the cohort ranged from 22 to 98 years (mean 76.4 ± 12 years). Approximately 53% of the study participants were men.

**Table 1:** Characteristics of the study participants at admission.

Variable	Overall (*n* = 370)	Quartile				*P*-value
		1 *N* = 99	2 *N* = 89	3 *N* = 91	4 *N* = 91	
Demographics						
Age, years	76.4 ± 12	75.9 ± 13.8	75.5 ± 11.6	78.1 ± 11	76.5 ± 11.1	0.456
Male sex	195 (52.7)	48 (48.5)	49 (55.1)	53 (58.2)	45 (49.5)	0.492
Medical history						
Obesity (BMI >30 kg/m^2^)	110 (29.7)	17 (17.2)	30 (33.7)	25 (27.5)	38 (41.8)	0.002
Hypertension	199 (53.8)	52 (52.5)	52 (58.4)	51 (56)	44 (48.3)	0.563
Dyslipidemia	75 (21.1)	18 (18.2)	19 (21.3)	18 (19.8)	20 (22)	0.937
Diabetes mellitus	126 (34.1)	21 (21.2)	28 (31.5)	37 (40.7)	40 (44)	0.003
Smoking	97 (26.2)	29 (29.3)	30 (33.7)	23 (25.3)	15 (16.5)	0.047
CKD	64 (17.3)	11 (11.1)	13 (14.6)	17 (18.7)	23 (25.3)	0.070
COPD	68 (18.4)	13 (13.1)	16 (18)	18 (19.8)	21 (23.1)	0.366
Anaemia^b^	43 (11.6)	7 (7.1)	7 (7.9)	13 (14.3)	16 (17.6)	0.075
CAD	157 (42.4)	36 (36.4)	36 (40.4)	41 (45.1)	44 (48.4)	0.371
Previous PCI	58 (15.7)	14 (14.1)	12 (13.5)	17 (18.7)	15 (16.5)	0.770
Previous CABG	70 (18.9)	15 (15.2)	15 (16.9)	12 (13.2)	28 (30.8)	0.010
Primary CMP	8 (2.2)	3 (3)	0 (0)	1 (1.1)	4 (4.4)	0.178
PAD	74 (20)	22 (22.2)	14 (15.7)	19 (20.9)	19 (20.9)	0.716
Arrhythmia	143 (38.6)	30 (30.3)	27 (30.3)	38 (41.8)	48 (52.7)	0.004
Atrial fibrillation	128 (34.6)	24 (24.2)	26 (29.2)	35 (38.5)	43 (47.3)	0.005
Known pulmonary hypertension	22 (5.9)	6 (6.1)	2 (2.2)	6 (6.6)	8 (8.8)	0.304
Triggers events for AHF						0.03
ACS	44 (11.9)	16 (16.2)	11 (12.4)	11 (12.1)	6 (6.6)	
AKD	4 (1.1)	0	0	0	4 (4.4)	
Anaemia	7 (1.9)	2 (2)	0	3 (3.3)	2 (2.2)	
Arrhythmias	84 (22.7)	20 (20.2)	22 (24.7)	20 (22)	22 (24.2)	
Cardiogenic shock	4 (1.1)	2 (2)	1 (1.1)	0	1 (1.1)	
Fluid overloading	101 (27.3)	20 (20.2)	19 (21.3)	27 (29.7)	35 (38.5)	
Hypertension	29 (7.8)	10 (10.1)	7 (7.9)	7 (7.7)	5 (5.5)	
Mechanical cause	35 (9.5)	8 (8.1)	6 (6.7)	11 (12.1)	10 (11)	
Non-adherence to medications/diet	4 (1.1)	2 (2)	0	0	2 (2.2)	
Pulmonary embolism	2 (0.5)	1 (1)	1 (1.1)	0	0	
Primary CMP	7 (1.9)	2 (2)	3 (3.4)	2 (2.2)	0	
Respiratory infection	29 (7.8)	13 (13.1)	10 (11.2)	4 (4.4)	2 (2.2)	
Others	20 (5.4)	3 (3)	9 (10.1)	6 (6.6)	2 (2.2)	
Chronic vs. *De Novo*						<0.001
Chronic HF	156 (42.2)	31 (31.3)	30 (33.7)	41 (45.1)	54 (59.3)	
De novo HF	214 (57.8)	68 (68.7)	59 (66.3)	50 (54.9)	37 (40.7)	
Clinical presentation						
Dyspnoea	358 (96.8)	96 (97)	84 (94.4)	90 (98.9)	88 (96.7)	0.513
Weight gain	102 (27.6)	11 (11.1)	18 (20.2)	28 (30.8)	45 (49.5)	0.000
Peripheral oedema	266 (71.9)	56 (56.6)	66 (74.2)	65 (71.4)	79 (86.8)	0.000
Chest Pain	82 (22.2)	23 (23.2)	17 (19.1)	29 (31.9)	13 (14.3)	0.037
Rhythm	(*n* = 366)	(*n* = 97)	(*n* = 88)	(*n* = 91)	(*n* = 90)	0.704
- SR	210 (57.4)	62 (63.9)	49 (55.7)	49 (53.8)	50 (54.3)	
- AF	131 (35.8)	27 (27.8)	33 (37.5)	35 (38.5)	36 (40.2)	
- Other	25 (6.8)	8 (8.2)	6 (6.8)	7 (7.7)	4 (4.3)	
BMI, kg/m^2^	27.9 ± 6.1	25.8 ± 4.9	27.7 ± 5.7	27.9 ± 6.1	30.3 ± 6.6	<0.001
SBP, mmHg	142.2 ± 30.9	144.2 ± 32.5	150 ± 29.1	141.9 ± 31.6	132.6 ± 27.7	0.002
DBP, mmHg	80 ± 19	82.3 ± 18.3	87.8 ± 19.5	79.4 ± 19.6	70.6 ± 14.3	<0.001
HR, bpm	95.2 ± 28	103 ± 33.2	100.9 ± 26.9	88.7 ± 23.8	87.7 ± 23.6	<0.001
O_2_ support	98 (26.5)	27 (27.3)	26 (29.2)	24 (26.4)	21 (23.1)	0.927
Chest x-ray congestion	284 (76.8)	79 (79.8)	68 (76.4)	71 (78)	66 (72.5)	0.783
Echo at admission						
LVEF	43.7 ± 16.4	44.8 ± 16	42.6 ± 17.1	43.3 ± 15.1	43.8 ± 16.4	0.869
Type of HF^a^						
- HFrEF	116 (40.1%)	28 (38.9%)	26 (40%)	28 (41.2%)	34 (40.5%)	0.935
- HFmrEF	55 (19%)	12 (16.7%)	15 (23.1%)	14 (20.6%)	14 (16.7%)	
- HFpEF	118 (40.8%)	32 (44.4%)	24 (36.9%)	26 (38.2%)	36 (42.9%)	
Cardiac devices						
ICD, CRT	32 (8.8%)	8 (8.2%)	5 (5.6%)	6 (6.7%)	13 (14.6%)	0.145
Laboratory values						
s-Cr, umol/L	111 (89–149)	103 (83–126)	103 (89–134)	120 (89–165)	137 (106–188)	<0.001
eGFR, ml/min	52 ± 29	52 ± 24	56 ± 26	49 ± 29	51 ± 37	0.517
Urea, mmol/L	9.15 (6.2–13.75	8 (5.48–9.35)	8.9 (6.2–10.6)	13.6 (6.95–14.9)	31.15 (6.83–52.4)	<0.001
K+, mmol/L	4.6 ± 1.1	4.5 ± 0.8	4.9 ± 1.4	4.7 ± 1	4.5 ± 0.9	0.053
Sodium, mmol/L	138 (135–141)	138 (137–141)	142 (139–143)	136 (135–139)	129 (122.5–132.5)	0.372
NT-proBNP (pmol/L)	662 [358–1,445]	691 [339–1,353]	609 [352–1,015]	743 [386–1,565]	672 [349–1,666]	0.515
Admission therapy						
Nitrates	85 (23)	17 (17.2)	13 (14.6)	30 (33)	25 (27.5)	0.009
MRA	52 (14.1)	9 (9.1)	8 (9)	17 (18.7)	18 (19.8)	0.035
HCT	45 (12.2)	13 (13.1)	14 (15.7)	5 (5.5)	13 (14.3)	0.152
Loop diuretics	190 (43.2)	29 (29.3)	36 (40.4)	54 (59.3)	71 (78)	<0.001
Mean daily loop diuretic dose, mg/day	120 [80–175]	57.5 [40–70]	105 [94–110]	140 [131–160]	240 [200–310]	<0.001
Index hospitalization therapy						
MRA	97 (26.2)	22 (22.2)	19 (21.3)	24 (26.4)	32 (35.2)	0.129
HCT	44 (11.9)	8 (8.1)	7 (7.9)	5 (5.5)	24 (26.4)	0.000
Nitrates	232 (62.7)	49 (49.5)	55 (61.8)	67 (73.6)	61 (67)	0.005
Inotropes	20 (5.4)	3 (3)	3 (3.4)	6 (6.6)	8 (8.8)	0.250

Data are presented as mean ± SD or number (percentage) of patients. CKD, chronic kidney disease; COPD, chronic pulmonary obstructive disease; SID, systemic inflammatory disease; CAD, coronary artery disease; CHF, chronic heart failure; PCI, percutaneous coronary intervention; CABG, coronary artery bypass graft surgery; CMP, cardiomyopathy; PAD, peripheral vascular disease; HVD, Heart valve disease; PH, pulmonary hypertension; ACS, acute coronary syndrome; AKD, acute kidney disease; HF, heart failure; SR, sinus rhythm; AF, atrial fibrillation; BMI, body mass index; SBP, systolic blood pressure; DBP, diastolic blood pressure; HR, heart rate; LVEF, left ventricular ejection fraction; s-Cr, serum creatinine; eGFR, estimated glomerular filtration rate; K+, potassium; NT-proBNP, N-terminal pro hormone B-type natriuretic peptide; ACE-I, angiotensin-converting enzyme inhibitor; ARB, angiotensin receptor blocker; MRA, mineralcorticoid receptor antagonist; HCT, hydrochlorothiazide. Mean daily loop diuretic dose, mg/day mean daily dose of Furosemide equivalent in the first week of hospitalisation.

^a^ The difference between percentages could be affected by missing data.

^b^ (Hb <7.5 mmol/L in women and <8.1 mmol/L in men).

The study population had several high-risk features including obesity, hypertension, dyslipidaemia, diabetes mellitus, smoking, coronary artery disease (CAD), peripheral artery disease, CKD and CHF. The most common triggers of AHF were fluid overload, arrhythmias, acute coronary syndromes, mechanical causes, hypertension and respiratory infection. At index admission, the majority of patients had signs and symptoms of congestion. The mean LVEF was 43.7 ± 16.1%. Patients treated with loop diuretics prior to index admission were 190 (51.4%) with the mean dosage of 120 [80–175] mg/day.

### Diuretic dose and patient characteristics

There were 99 patients (26.7%) in the first quartile, with mean daily furosemide dose equivalence of 0–80 mg/day; 89 patients (24.1%) in the second quartile, with dose equivalence of 81–120 mg/day; 91 patients (24.6%) in the third quartile, with dose equivalence of 121–175 mg/day; and 91 patients (24.6%) in the fourth quartile, with dose equivalence >175 mg/day. Differences in patients’ characteristics among the loop diuretic quartiles are depicted in Table 1. Patients receiving higher doses of loop diuretics more often had diabetes mellitus, previous heart failure, arrhythmias, primary cause of admission was more often fluid overload, had more prior history of CABG, experienced more weight gain, and had more signs of right ventricular fluid overload, higher BMI and lower blood pressure and heart rate, higher admission s-Cr, urea and NT-proBNP. They were treated prior to admission more often with loop diuretic at higher doses and mineralcorticoid receptor antagonist (MRA). They also received more often thiazides during hospitalisation and nitrates. In addition, there was a more important prevalence of smokers in the second quartile, and chest pain at presentation and nitrates were prevalent in the third quartile (Supplementary Table S1).

### Relation between diuretic dose and primary outcome

**Figure 1:**
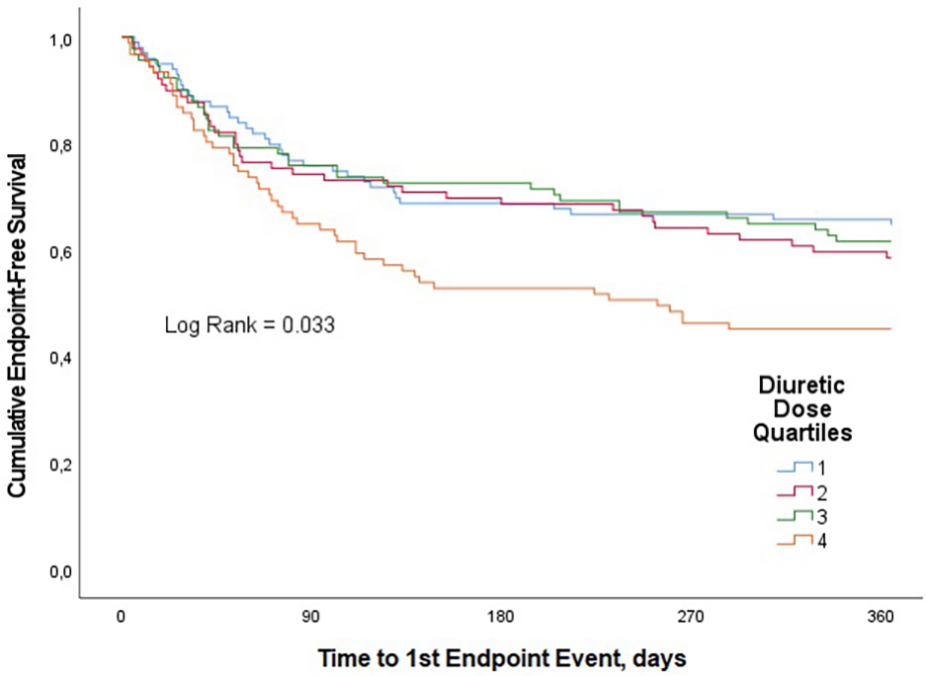
Kaplan–Meier curves for the clinical composite end point of CV mortality or CV rehospitalization for loop diuretic dose quartiles in patients with AHF over one-year follow-up.

**Figure 2:**
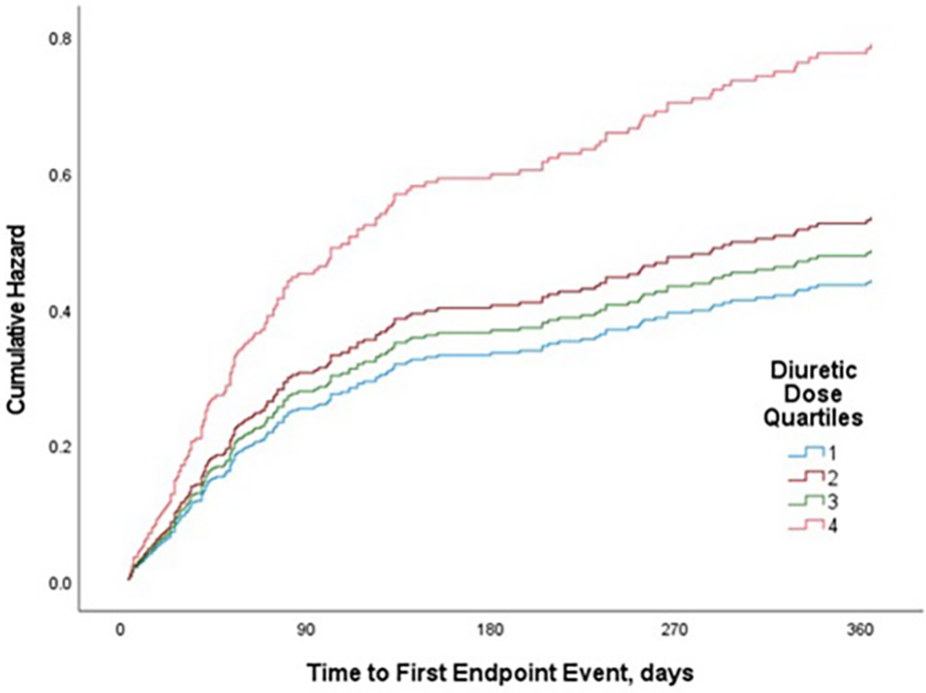
Cox proportional hazard model (univariate): risk of composite end point of CV mortality or CV rehospitalization for loop diuretic dose quartiles in patients with AHF over one-year follow-up.

A total of 157 patients (42.4%) had a CV rehospitalization or died with CV causes within the one-year follow-up period. CV rehospitalization or CV mortality estimates at 1 year were 35.4%, 41.6%, 38.5%, and 54.9% for quartiles 1, 2, 3, and 4, respectively (*p* = 0.033) (Figure 1). In univariate analysis, this composite endpoint was significantly lower in the first three quartiles as compared to the quartile with the highest loop diuretic dose [first quartile, OR 0.45, 95% confidence interval (CI) 0.25–0.8; second quartile, OR 0.58, 95% CI 0.32–1.1 and third quartile, OR 0.51, 95% CI 0.–0.92; *p* = 0.040], whereas between the first three quartiles, there was no statistically significant difference. In univariable Cox proportional hazard model, highest diuretic dose quartile was associated with an increased risk of primary outcome (HR 1.62, 95% CI 1.05–2.27; *p*-value = 0.005) compared to the other quartiles (Figure 2).

After adjustment for age, gender, diabetes mellitus, smoking, obesity, hypertension, arrhythmia, previous CHF, admission SBP ≤90 mmHg, admission creatinine, admission urea, admission sodium and admission NT-proBNP, the association between loop diuretic dosage and the primary end point disappeared (Table 2).

**Table 2:** Association between quartiles of daily mean dose of furosemide equivalents and clinical end points.

	Overall *N* = 370	Quartile				*P*-value
		1 ≤80 mg *N* = 99	2 81–120 mg *N* = 89	3 121–175 mg *N* = 91	4 >175 mg *N* = 91	
One-year CV mortality or CV rehospitalizations	157 (42.4)	35 (35.4)	37 (41.6)	35 (38.5)	50 (54.9)	***0.037***
		0.45 (0.25–0.8)	0.58 (0.32–1.1)	0.51 (0.28–0.92)	Reference	***0.040***
Multivariate analysis^a^		0.54 (0.25–1.15)	0.85 (0.40–1.19)	0.6 (0.330–1.22)	Reference	0.285
Multivariate analysis (only age/gender)^b^		0.44 (0.25–0.79)	0.58 (0.32–1.04)	0.53 (0.29–0.96)	Reference	0.040
One-year CV mortality	62 (16.8)	9 (9.1)	9 (10.1)	19 (20.9)	25 (27.2)	***0.001***
		0.26 (0.12–0.6)	0.3 (0.13–0.68)	0.7 (0.35–1.4)	Reference	***0.002***
Multivariate analysis^a^		0.45 (0.16–1.26)	0.41 (0.13–1.25)	0.94 (0.4–2.22)	Reference	0.232
Multivariate analysis (only age/gender)^b^		0.26 (0.12–0.60)	0.30 (0.13–0.68)	0.68 (0.34–1.34)	Reference	0.002
3-months CV mortality or CV rehospitalizations	102 (27.6)	24 (24.2)	23 (25.8)	22 (24.2)	33 (36.3)	0.199
		0.56 (0.3–1.05)	0.61 (0.32–1.16)	0.56 (0.3–1.07)	Reference	0.204
3-months CV mortality	38 (10.3)	8 (8.1)	6 (6.7)	11 (12.1)	13 (14.3)	0.304
		0.53 (0.21–1.34)	0.43 (0.16–1.2)	0.83 (0.35–1.95)	Reference	0.317
3-months CV rehospitalizations	67 (18.1)	17 (17.2)	17 (19.1)	13 (14.3)	20 (22)	0.586
		0.74 (0.36–1.51)	0.84 (0.41–1.73)	0.59 (0.27–1.28)	Reference	0.590
One-year CV rehospitalizations	106 (28.6)	27 (27.3)	29 (32.6)	20 (22)	30 (33)	0.295
		0.75 (0.4–1.4)	0.97 (0.51–1.8)	0.56 (0.29–1.1)	Reference	0.299
In-hospital CV mortality	21 (5.7)	2 (2)	4 (4.5)	5 (5.5)	10 (11)	0.057
		0.17 (0.04–0.78)	0.38 (0.12–1.26)	0.47 (0.15–1.44)	Reference	0.088
WRF	156 (42.2)	39 (39.4)	41 (46.1)	43 (47.3)	33 (36.3)	0.503
		1.23 (0.67–2.24)	1.4 (0.77–2.55)	1.56 (0.86–2.85)	Reference	0.505
Hypokalaemia	117 (31.6)	18 (18.2)	36 (40.4)	29 (31.9)	34 (37.4)	***0.005***
		0.37 (0.19–0.72)	1.14 (0.63–2.07)	0.78 (0.43–1.45)	Reference	***0.006***
Hyperkalaemia	126 (34.1)	29 (29.3)	38 (42.7)	33 (36.3)	26 (28.6)	0.146
		1.0 (0.55–1.94)	1.86 (1.0–3.46)	1.42 (0.76–2.66)	Reference	0.150
Dyspnoea relief	335/358 (93.6)	91/96 (94.8)	80/84 (95.2)	85/90 (94.4)	79/88 (89.8)	0.997
		1.15 (0.28–4.76)	1.01 (0.25–4.19)	1.08 (0.26–4.45)	Reference	0.997
Length of hospital stay	13 ± 14.1	8 (6–15)	8 (6–13.5)	9 (6–15)	13 (8–20)	<0.001
Weight loss	−5.8 ± 5.2	−3.4 ± 3.6	−5.4 ± 3.9	−5.2 ± 4.7	−8.3 ± 6.5	***0.000***

Data are presented as mean ± SD or number (percentage) of patients or OR (95% CI). CV, cardiovascular; WRF, worsening of renal function.

^a^ Multivariate analysis included: age, male sex, obesity, diabetes mellitus, smoking, hypertension, arrhythmia, chronic heart failure (CHF), admission systolic blood pressure (SBP) ≤90 mmHg, admission creatinine, admission urea, admission sodium and admission NT-proBNP.

^b^ Multivariate analysis included: age and gender.

Bold values indicate significant *p*-values.

Supplementary Table S2 lists the characteristics for patients with the primary end point compared with patients without it.

### Relation between diuretic dose and secondary outcomes

**Figure 3:**
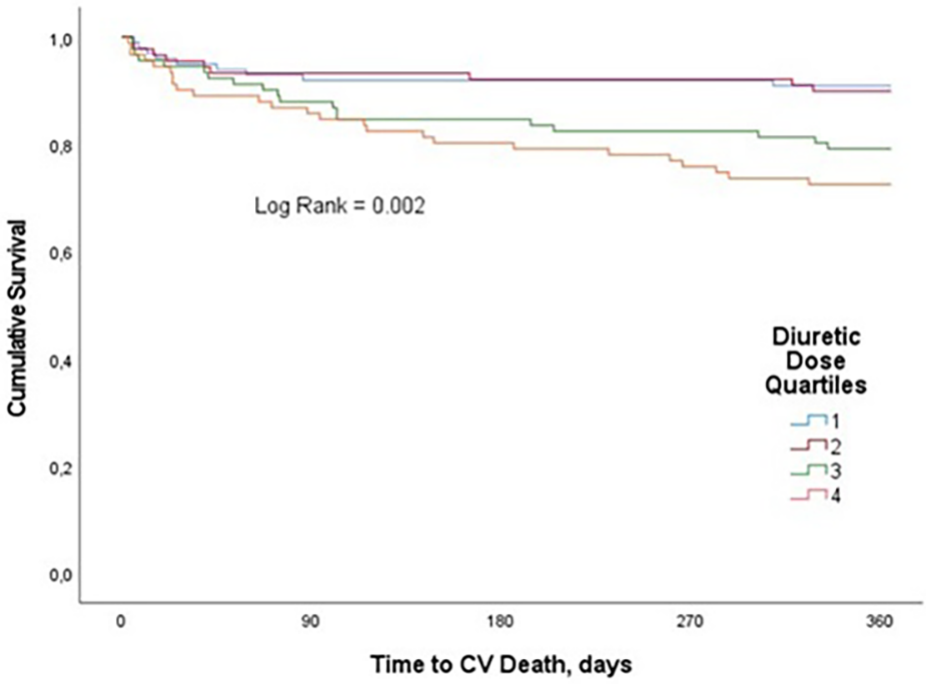
Kaplan–Meier curves for the clinical end point of CV mortality for loop diuretic dose quartiles in patients with AHF over one-year follow-up.

We observed 62 CV deaths (16.8% of study participants) during the one-year follow-up period. In-hospital CV death occurred in 21 (5.7%) patients. Patients in the highest diuretic dose quartile were found to have significantly worse survival compared with patients in the lowest quartile. CV mortality estimates at 1 year were 9.1%, 10.1%, 20.9% and 27.2% for quartiles 1, 2, 3, and 4, respectively (*p* = 0.002) (Figure 3). In univariate analysis, compared with the fourth quartile, increasing loop diuretic dose quartiles were associated with lower CV mortality in the first two quartiles (first quartile, OR 0.26, 95% CI 0.12–0.6; second quartile, OR 0.3, 95% CI 0.13–0.68; and third quartile, OR 0.7, 95% CI 0.35–1.4; *p* = 0.002).

After adjustment for age, male sex, obesity, diabetes mellitus, smoking, hypertension, arrhythmia, CHF, admission SBP ≤90 mmHg, admission creatinine, admission urea, admission sodium and admission NT-proBNP, no statistically significant association was found between loop diuretic dosage and one-year CV mortality (Table 2).

Non-survivors for CV causes had more often CKD, CAD, previous HF, pulmonary hypertension, and previous CABG, and experienced lower blood pressure, heart rate, eGFR and sodium, higher s-Cr and urea. Furthermore, they were more often treated prior to admission with loop diuretic, beta-blocker and MRA, and during the index hospitalisation they received more often MRA and inotropes and higher doses of loop diuretic (Supplementary Table S3).

Subjects in the highest diuretic dose quartile had significantly more often hypokalaemia (serum potassium <3.5 mmol/L) compared with patients in the lowest quartile, whereas there were no statistically significant differences between groups in terms of hyperkalaemia (serum potassium >5 mmol/L).

Moreover, there were no significant between-groups differences for a variety of secondary endpoints (Table 2): CV rehospitalization during the one-year follow-up period, CV mortality or rehospitalizations and CV mortality during the three-month follow-up period, in-hospital CV mortality, WRF and dyspnoea relief at discharge. Patients in the highest diuretic dose quartile were found to have significantly longer hospitalizations and greater weight loss during the index hospitalization compared with patients in the lowest one.

## Discussion

Despite the widespread loop diuretics strategy, their appropriate use remains challenging given the limited evidence supporting their efficacy, safety (especially WRF), the optimal dosage and the relative prognostic impact (13). The current study demonstrates that patients requiring higher loop diuretic doses had more comorbidities, more often a history of CHF and impaired clinical conditions including more congestion and lower blood pressure. The potentially negative impact on outcome of higher doses of loop diuretics to recompensate can only be seen long-term and disappears completely in multivariable analysis. This suggests that the need of more intensified diuretic therapy is an indicator of severity of AHF to completely decongest and of co-morbidities but is not harmful *per se*. Thus, the report of this study supports the findings of the DOSE study in a real-world population that was older and had more co-morbidities.

Other observational studies have found that higher doses may have harmful effects, possibly due to activation of the renin-angiotensin and sympathetic nervous system, or electrolyte disturbance (14, 15). Still, it must be underlined that such observations are confounded by the fact that more severely ill patients require higher doses of diuretic, particularly when the urine output of sodium and water is insufficient. Indeed, our data supports the idea that higher doses of loop diuretics are a consequence of more advanced HF rather than a cause of poor outcome. Therefore, these should be considered as a marker instead of a mechanism of poor outcomes (15, 16). Patients with higher loop diuretic dose also more often required the addition of thiazide-type diuretics. These drugs are prescribed in less responsive patients to address loop diuretic resistance because they might overcome the escape phenomenon due to activation of the renin-angiotensin system (RAS) and sympathetic system and sodium reabsorption by more distal sodium transporters (17). Administration of high loop diuretics doses, particularly in combination with thiazides, may lead to electrolyte imbalances (such hypokalaemia, hyponatremia, and hypomagnesemia), which might exacerbate cardiac arrhythmias and increase the risk of sudden cardiac death (14). It may be speculated that the daily monitoring of electrolytes during decongestion or the occurrence of loop diuretic resistance that limited the excessive loss of fluid and electrolyte in our study population could have contributed to the lack of increased in-hospital CV mortality in the patients receiving higher doses.

WRF during an AHF hospitalization is common ranging from 20 to 40% (18), but not always clinically relevant, especially when it is associated with appropriate decongestion, diuresis and haemoconcentration (1). CKD is a well-recognized risk factor of adverse outcomes in AHF (7). However, the latter was not confirmed for acute renal dysfunction. On one hand, several studies reported that the association between the decline in kidney function and AHF treatment is not related with adverse outcomes (8, 19); on the other hand, the association with poor prognosis (20) through several mechanisms such as inflammation, oxidative stress, impaired hydrosaline homeostasis, and diuretic resistance (21) was demonstrated in other studies. WRF occurred in approximately 40% of our study population, but it was not related to loop diuretic dose. This finding is extremely relevant since it overcomes the prejudice regarding the fear of decreasing renal plasma flow and deterioration of kidney function. WRF is frequently interpreted in clinical practice as a decrease in effective circulating volume, prompting physicians to reduce loop diuretic therapy based on the often-false assumption that further decongestion might result in renal tubular damage (22).

Furthermore, the association between WRF and outcomes might be influenced by other factors. Firstly, patients who experience WRF have often a higher disease severity. Secondly, they are less responsive to AHF therapies and, finally, they are intrinsically at greater risk of adverse events independent of the renal dysfunction (8). WRF alone is not an independent determinant of outcomes in patients with AHF, but it has an additive prognostic value when it occurs in patients with persistent signs of congestion (19). This implies that the renal function impairment is more dependent on venous congestion rather than on impairment of cardiac output (23). The limited impact of aggressive decongestion with higher doses of loop diuretics on renal function is supported by the findings of this study, which is in line with very recent data on the addition of acetazolamide (24) and thiazide (25) to rapidly decongest patients with ADHF. The main goal should therefore be rapid and effective decongestion followed by establishment of standard therapy of chronic HF (22, 26). Our data supports this recommendation also in an elderly all-comers population of ADHF, in which renal function is generally poorer and treating physicians more reluctant to use higher diuretic doses. In fact, despite the use of diuretics, 40% of patients are discharged with unresolved congestion in clinical practice, leading to increased rehospitalization and higher mortality rates (27).

## Study limitations

The present study has the following limitations. First, the retrospective nature of this study does not allow to certainly define the causal relationship of the investigated variables.

Second, WRF definition depends on s-Cr, which is primarily a marker of glomerular filtration with a slow kinetics and does not recognize renal tubular injury in the absence of a significant reduction in eGFR (28).

Third, weight loss, as indicator of decongestion and diuretic response in the absence of diuresis data, is influenced by factors other than fluid balance. This may be inaccurate even in the best of circumstances, may be a poor predictor of euvolemia and has a weak correlation with net urine output in AHF clinical trials (29–31). Furthermore, data on predischarge NT-proBNP and delta changes during hospitalisation were available for a very small number of patients, and therefore could not be analysed as a marker of decongestion and clinical improvement. Moreover, a comprehensive haemodynamic phenotyping to proof complete decongestion at discharge (central venous pressure and pulmonary artery wedge pressure) was not collected in the present study. Finally, the limited number of patients included might have contributed to the lack of prognostic impact of diuretic therapy during first months, which is in some contrast to earlier studies.

## Conclusion

The highest diuretic dose was associated with a significant increase in CV mortality and in the composite endpoint of CV mortality or CV rehospitalization over the one-year follow-up period, while there was no association between higher doses and outcomes at 3 months. In addition, poor long-term outcome was no longer seen in multivariable analysis. Furthermore, higher diuretic dosage was not associated with a significant worsening in renal function. These findings suggest that higher doses of loop diuretics are not harmful but necessary to manage congestion in patients with advanced HF and worse admission clinical conditions, explaining the poor outcome at 1 year. Our data are in line with recent finding that aggressive decongestion may result in better outcome (24, 25) and suggests that such a treatment regimen is also applicable in an elderly all-comer population with ADHF.

## Data Availability

The raw data supporting the conclusions of this article will be made available by the authors, without undue reservation.
