# Supplementary material for: Impact of loop diuretic dosage in a population of patients with acute heart failure: a retrospective analysis
Source: Front Cardiovasc Med. 2023 Nov 23;10:1267042. doi: 10.3389/fcvm.2023.1267042 (PMC10701382; doi:10.3389/fcvm.2023.1267042)
Supplement: Supplementary file 1 [file Datasheet1.docx]

# **Supplementary data**

**Supplementary methods**

The specific AHF trigger was identified by the physician (mainly, cardiologist or cardio-intensivist) who admitted the patient to the cardiology department or Intensive Care Unit.

The trigger was defined as follows:

- ACS: patients presenting with recent changes in clinical symptoms or signs, with or without changes on 12-lead electrocardiogram and with or without acute elevations in cardiac troponin concentrations.

- AKD: acute kidney disease describes acute or subacute damage and/or loss of kidney function (>for a duration of between 7 and 90 days)

- Anaemia: new onset of Hb<7.5 mmol/l in women or <8.1 mmol/l in men

- Arrhythmias

- Cardiogenic shock: SBP<90mmHg for>30min or support to maintain SBP>=90mmHg and end-organ hypoperfusion (urine output<0,5 ml/kg/h or cold extremities)

- Fluid Overloading: dyspnoea and presence of clinical signs suggesting fluid overload (peripheral oedema and/or pulmonary oedema and/or pulmonary rales and/or weight gain and/or jugular vein distension)

- Hypertension: rapid onset of pulmonary congestion in the setting of a systolic blood pressure >140 mm Hg

- Mechanical cause: new onset of moderate/severe valvular disease (either regurgitation or stenosis)

- Non-adherence medication/ diet

- Pulmonary embolism: pulmonary embolism demonstrated with thoracic angioCT

- Primary cardiomyopathy

- Respiratory infection: any infectious disease of the upper or lower respiratory tract associated with systemic inflammation (increased white blood cell count and/or C-reactive protein)

# **Tables**

## **Table S1. Characteristics of the study participants at admission(full table)**

| **Variable** |  | **Quartile** | | | | **P Value** |
| --- | --- | --- | --- | --- | --- | --- |
|  | **Overall (n=370)** | **1**  **N=99** | **2**  **N=89** | **3**  **N=91** | **4**  **N=91** |  |
| **Demographics** |  |  |  |  |  |  |
| Age, years | 76.4±12 | 75.9±13.8 | 75.5±11.6 | 78.1±11 | 76.5±11.1 | 0.456 |
| Male sex | 195 (52.7) | 48 (48.5) | 49 (55.1) | 53 (58.2) | 45 (49.5) | 0.492 |
| **Medical History** |  |  |  |  |  |  |
| Obesity (BMI>30 kg/m2) | 110 (29.7) | 17(17.2) | 30 (33.7) | 25 (27.5) | 38 (41.8) | 0.002 |
| Hypertension | 199 (53.8) | 52 (52.5) | 52 (58.4) | 51 (56) | 44 (48.3) | 0.563 |
| Dyslipidemia | 75 (21.1) | 18 (18.2) | 19 (21.3) | 18 (19.8) | 20 (22) | 0.937 |
| Diabetes mellitus | 126 (34.1) | 21 (21.2) | 28 (31.5) | 37 (40.7) | 40 (44) | *0.003* |
| Smoking | 97 (26.2) | 29 (29.3) | 30 (33.7) | 23 (25.3) | 15 (16.5) | *0.047* |
| CKD | 64 (17.3) | 11 (11.1) | 13 (14.6) | 17 (18.7) | 23 (25.3) | 0.070 |
| COPD | 68 (18.4) | 13 (13.1) | 16 (18) | 18 (19.8) | 21 (23.1) | 0.366 |
| Cancer | 67 (18.1) | 20 (20.2) | 16 (18) | 16 (17.6) | 15 (16.5) | 0.914 |
| Anaemia^#^ | 43 (11.6) | 7 (7.1) | 7 (7.9) | 13 (14.3) | 16 (17.6) | 0.075 |
| CAD | 157 (42.4) | 36 (36.4) | 36 (40.4) | 41 (45.1) | 44 (48.4) | 0.371 |
| Previous PCI | 58 (15.7) | 14 (14.1) | 12 (13.5) | 17 (18.7) | 15 (16.5) | 0.770 |
| Previous CABG | 70 (18.9) | 15 (15.2) | 15 (16.9) | 12 (13.2) | 28 (30.8) | *0.010* |
| Primary CMP | 8 (2.2) | 3 (3) | 0 (0) | 1 (1.1) | 4 (4.4) | 0.178 |
| PAD | 74 (20) | 22 (22.2) | 14 (15.7) | 19 (20.9) | 19 (20.9) | 0.716 |
| Arrhythmia | 143 (38.6) | 30 (30.3) | 27 (30.3) | 38 (41.8) | 48 (52.7) | *0.004* |
| Atrial fibrillation | 128 (34.6) | 24 (24.2) | 26 (29.2) | 35 (38.5) | 43 (47.3) | *0.005* |
| HVD | 99 (26.8) | 24 (24.2) | 22 (24.7) | 23 (25.3) | 30 (33.3) | 0.459 |
| Known pulmonary hypertension | 22 (5.9) | 6 (6.1) | 2 (2.2) | 6 (6.6) | 8 (8.8) | 0.304 |
| Stroke/TIA | 68 (18.4) | 22 (22.2) | 14 (15.7) | 15 (16.5) | 17 (18.7) | 0.656 |
| **Triggers events for AHF** | |  |  |  |  | *0.03* |
| ACS | 44 (11.9) | 16 (16.2) | 11 (12.4) | 11 (12.1) | 6 (6.6) |  |
| AKD | 4 (1.1) | 0 | 0 | 0 | 4 (4.4) |  |
| Anaemia | 7 (1.9) | 2 (2) | 0 | 3 (3.3) | 2 (2.2) |  |
| Arrhythmias | 84 (22.7) | 20 (20.2) | 22 (24.7) | 20 (22) | 22 (24.2) |  |
| Cardiogenic shock | 4 (1.1) | 2 (2) | 1 (1.1) | 0 | 1 (1.1) |  |
| Fluid Overloading | 101 (27.3) | 20 (20.2) | 19 (21.3) | 27 (29.7) | 35 (38.5) |  |
| Hypertension | 29 (7.8) | 10 (10.1) | 7 (7.9) | 7 (7.7) | 5 (5.5) |  |
| Mechanical cause | 35 (9.5) | 8 (8.1) | 6 (6.7) | 11 (12.1) | 10 (11) |  |
| Non-adherence to medication or diet | 4 (1.1) | 2 (2) | 0 | 0 | 2 (2.2) |  |
| Pulmonary embolism | 2 (0.5) | 1 (1) | 1 (1.1) | 0 | 0 |  |
| Primary CMP | 7 (1.9) | 2 (2) | 3 (3.4) | 2 (2.2) | 0 |  |
| Respiratory infection | 29 (7.8) | 13 (13.1) | 10 (11.2) | 4 (4.4) | 2 (2.2) |  |
| Toxic damage | 3 (0.8) | 1 (1) | 1 (1.1) | 1 (1.1) | 0 |  |
| Others | 17 (4.6) | 2 (2) | 8 (9) | 5 (5.5) | 2 (2.2) |  |
| **Setting** |  |  |  |  |  | 0.742 |
| -In Hospital | 14 (3.8) | 5 (5.1) | 4 (4.5) | 2 (2.2) | 3 (3.3) |  |
| -Out Hospital | 356 (96.2) | 94 (94.9) | 85 (95.5) | 89 (97.8) | 88 (96.7) |  |
| **Chronic vs De Novo** | |  |  |  |  | *<0.001* |
| Chronic HF | 156 (42.2) | 31 (31.3) | 30 (33.7) | 41 (45.1) | 54 (59.3) |  |
| De novo HF | 214 (57.8) | 68 (68.7) | 59 (66.3) | 50 (54.9) | 37 (40.7) |  |
| **Clinical presentation** | |  |  |  |  |  |
| Dyspnoea | 358 (96.8) | 96 (97) | 84 (94.4) | 90 (98.9) | 88 (96.7) | 0.513 |
| Orthopnoea | 169 (45.7) | 39 (39.4) | 42 (47.2) | 41 (45.1) | 47 (51.6) | 0.361 |
| Weight gain | 102 (27.6) | 11 (11.1) | 18 (20.2) | 28 (30.8) | 45 (49.5) | *0.000* |
| Jugular venous distension * | 166 (44.9) | 30 (30.3) | 46 (51.7) | 43 (47.3) | 47 (51.6) | *0.001* |
| Fatigue | 33 (8.9) | 10 (10.1) | 7 (7.9) | 10 (11) | 6 (6.6) | 0.705 |
| Cold extremities | 10 (2.7) | 2 (2) | 4 (4.5) | 0 | 4 (4.4) | 0.190 |
| Pulmonary rales | 268 (72.4) | 71 (71.7) | 60 (67.4) | 69 (75.8) | 68 (74.7) | 0.690 |
| Peripheral oedema | 266 (71.9) | 56 (56.6) | 66 (74.2) | 65 (71.4) | 79 (86.8) | *0.000* |
| Chest Pain | 82 (22.2) | 23 (23.2) | 17 (19.1) | 29 (31.9) | 13(14.3) | *0.037* |
| Rhythm | (n=366) | (n=97) | (n=88) | (n=91) | (n=90) | 0.704 |
| -SR | 210 (57.4) | 62 (63.9) | 49 (55.7) | 49 (53.8) | 50 (54.3) |  |
| -AF | 131 (35.8) | 27 (27.8) | 33 (37.5) | 35 (38.5) | 36 (40.2) |  |
| -Other | 25 (6.8) | 8 (8.2) | 6 (6.8) | 7 (7.7) | 4 (4.3) |  |
| BMI, kg/m^2^ | 27.9±6.1 | 25.8±4.9 | 27.7±5.7 | 27.9±6.1 | 30.3±6.6 | *<0.001* |
| SBP, mmHg | 142.2±30.9 | 144.2±32.5 | 150±29.1 | 141.9±31.6 | 132.6±27.7 | *0.002* |
| DBP, mmHg | 80±19 | 82.3±18.3 | 87.8±19.5 | 79.4±19.6 | 70.6±14.3 | *<0.001* |
| HR, bpm | 95.2±28 | 103±33.2 | 100.9±26.9 | 88.7±23.8 | 87.7±23.6 | *<0.001* |
| O_2_ support | 98 (26.5) | 27 (27.3) | 26 (29.2) | 24 (26.4) | 21 (23.1) | 0.927 |
| Chest X-ray congestion | 284 (76.8) | 79 (79.8) | 68 (76.4) | 71 (78) | 66 (72.5) | 0.783 |
| **Echo** |  |  |  |  |  |  |
| LVEF | 43.7±16.4 | 44.8±16 | 42.6±17.1 | 43.3±15.1 | 43.8±16.4 | 0.869 |
| Type of HF*  HFrEF  HFmrEF  HFpEF | 116 (40.1%)  55 (19%)  118 (40.8%) | 28 (38.9%)  12 (16.7%)  32 (44.4%) | 26 (40%)  15 (23.1%)  24 (36.9%) | 28 (41.2%)  14 (20.6%)  26 (38.2%) | 34 (40.5%)  14 (16.7%)  36 (42.9%) | 0.935 |
| **Laboratory values** |  |  |  |  |  |  |
| s-Cr, umol/l | 111 (89-149) | 103 (83-126) | 103 (89-134) | 120 (89-165) | 137 (106-188) | *<0.001* |
| eGFR, ml/min | 52±29 | 52±24 | 56±26 | 49±29 | 51±37 | 0.517 |
| Urea, mmol/l | 9.15 (6.2-13.75 | 8 (5.48-9.35) | 8.9 (6.2-10.6) | 13.6 (6.95-14.9) | 31.15 (6.83-52.4) | *<0.001* |
| K+, mmol/l | 4.6±1.1 | 4.5±0.8 | 4.9±1.4 | 4.7±1 | 4.5±0.9 | 0.053 |
| Sodium, mmol/l | 138 (135-141) | 138 (137-141) | 142 (139-143) | 136 (135-139) | 129 (122.5-132.5) | 0.372 |
| NT-proBNP (pmol/l) | 662 [358-1445] | 691 [339-1353] | 609 [352-1015] | 743 [386-1565] | 672 [349-1666] | 0.515 |
| Log NT-proBNP | 2.84±0.45 | 2.82±0.43 | 2.77±0.43 | 2.87±0.44 | 2.89±0.5 | 0.350 |
| **Admission therapy** | |  |  |  |  |  |
| Beta-blocker | 239 (64.6) | 58 (58.6) | 60 (67.4) | 56 (61.5) | 65 (71.4) | 0.172 |
| ACE-I | 115 (31.1) | 28 (28.3) | 22 (24.7) | 31 (34.1) | 34 (37.4) | 0.201 |
| ARB | 95 (25.7) | 26 (26.3) | 26 (29.2) | 20 (22) | 23 (25.3) | 0.761 |
| Nitrates | 85 (23) | 17 (17.2) | 13 (14.6) | 30 (33) | 25 (27.5) | *0.009* |
| MRA | 52 (14.1) | 9 (9.1) | 8 (9) | 17 (18.7) | 18 (19.8) | *0.035* |
| HCT | 45 (12.2) | 13 (13.1) | 14 (15.7) | 5 (5.5) | 13 (14.3) | 0.152 |
| Loop diuretics | 190 (43.2) | 29 (29.3) | 36 (40.4) | 54 (59.3) | 71 (78) | *<0.001* |
| Mean daily loop diuretic dose, mg/day | 120 [80-175] | 57.5 [40-70] | 105 [94-110] | 140 [131-160] | 240 [200-310] | *<0.001* |
| **Index hospitalization therapy** | |  |  |  |  |  |
| Beta-blocker | 299 (80.8) | 84 (84.8) | 75 (84.3) | 71 (78) | 69 (75.8) | 0.303 |
| ACE-I | 188 (50.8) | 57 (57.6) | 46 (51.7) | 45 (49.5) | 40 (44) | 0.306 |
| ARB | 88 (23.8) | 22 (22.2) | 26 (29.2) | 20 (22) | 20 (22) | 0.592 |
| MRA | 97 (26.2) | 22 (22.2) | 19 (21.3) | 24 (26.4) | 32 (35.2) | 0.129 |
| HCT | 44 (11.9) | 8 (8.1) | 7 (7.9) | 5 (5.5) | 24 (26.4) | *0.000* |
| Nitrates | 232 (62.7) | 49 (49.5) | 55 (61.8) | 67 (73.6) | 61 (67) | *0.005* |
| Inotropes | 20 (5.4) | 3 (3) | 3 (3.4) | 6 (6.6) | 8 (8.8) | 0.250 |

Data are presented as mean ± SD or number (percentage) of patients. CKD = chronic kidney disease; COPD = chronic pulmonary obstructive disease; SID = systemic inflammatory disease; CAD = coronary artery disease; CHF = chronic heart failure; PCI = percutaneous coronary intervention; CABG = coronary artery bypass graft surgery; CMP = cardiomyopathy; PAD = peripheral vascular disease; HVD = Heart valve disease; PH = pulmonary hypertension; ACS = acute coronary syndrome; AKD = Acute Kidney Disease; HF = heart failure; SR = sinus rhythm; AF = atrial fibrillation; BMI = body mass index; SBP = systolic blood pressure; DBP = diastolic blood pressure; HR = heart rate; LVEF = left ventricular ejection fraction; s-Cr = serum creatinine; eGFR = estimated glomerular filtration rate; K+ = potassium;  NT-proBNP = N-terminal pro hormone B-type natriuretic peptide; ACE-I = angiotensin-converting enzyme inhibitor; ARB = angiotensin receptor blocker; MRA = mineralcorticoid receptor antagonist; HCT = hydrochlorothiazide. Mean daily loop diuretic dose, mg/day mean daily dose of Furosemide equivalent in the first week of hospitalisation. * The difference between percentages could be affected by missing data.# Hb<7.5 mmol/l in women or <8.1 mmol/l in men.

## **Table S2. Characteristics of patients with and without primary end point (full table) .**

| **Variable** | **No primary end point**  **N=213** | **Primary end point**  **N=157** | **p Value** |
| --- | --- | --- | --- |
| **Demographics** |  |  |  |
| Age, years | 77.4±11.6 | 75.1±12.3 | 0.063 |
| Male sex | 112 (52.6) | 83 (52.9) | 0.957 |
| **Medical History** |  |  |  |
| Obesity (BMI>30 kg/m2) | 69 (32.4) | 41 (26.1) | 0.191 |
| Hypertension | 119 (55.9) | 80 (51) | 0.457 |
| Dyslipidemia | 42 (19.7) | 33 (21) | 0.692 |
| Diabetes mellitus | 69 (32.4) | 57 (36.3) | 0.425 |
| Smoking | 61 (28.6) | 36 (22.9) | 0.210 |
| CKD | 31 (14.6) | 33 (21) | 0.102 |
| COPD | 38 (17.8) | 30 (19.1) | 0.772 |
| Cancer | 43 (20.2) | 24 (15.3) | 0.229 |
| Anaemia^#^ | 23 (10.8) | 20 (12.7) | 0.550 |
| CAD | 80 (37.6) | 77 (49) | *0.030* |
| Previous PCI | 31 (14.6) | 27 (17.2) | 0.502 |
| Previous CABG | 32 (15) | 38 (24.2) | *0.026* |
| Primary CMP | 3 (1.4) | 5 (3.2) | 0.242 |
| PAD | 46 (21.6) | 28 (17.8) | 0.363 |
| Arrhythmia | 79 (37.1) | 64 (40.8) | 0.473 |
| Atrial fibrillation | 69 (32.4) | 59 (37.6) | 0.300 |
| HVD | 58 (27.2) | 41 (26.1) | 0.839 |
| Known Pulmonary Hypertension | 10 (4.7) | 12 (7.6) | 0.230 |
| Stroke/TIA | 39 (18.3) | 29 (18.5) | 0.968 |
| **Triggers events for AHF** |  |  | 0.089 |
| -ACS | 24 (11.3) | 20 (12.7) |  |
| -AKD | 2 (0.9) | 2 (1.3) |  |
| -Anaemia | 6 (2.8) | 1 (0.6) |  |
| -Arrhythmias | 53 (24.9) | 31 (19.7) |  |
| -CS | 2 (0.9) | 2 (1.3) |  |
| -Fluid Overloading | 61 (28.6) | 40 (25.5) |  |
| -Hypertension | 15 (7) | 14 (8.9) |  |
| -Mechanical cause | 15 (7) | 20 (12.7) |  |
| -Non-adherence to medication or diet | 1 (0.5) | 3 (1.9) |  |
| -PE | 1 (0.5) | 1 (0.6) |  |
| -Primary CMP | 1 (0.5) | 6 (3.8) |  |
| -Respiratory infection | 21 (9.9) | 8 (5.1) |  |
| -Toxic damage | 3 (1.4) | 0 |  |
| -Others | 8 (3.8) | 9 (5.7) |  |
| **Setting** |  |  | 0.105 |
| -In Hospital | 11 (5.2) | 3 (1.9) |  |
| -Out Hospital | 202 (94.8) | 154 (98.1) |  |
| **Chronic vs De Novo** |  |  | *0.021* |
| -CHF | 79 (37.1) | 77 (49) |  |
| -De novo HF | 134 (62.9) | 80 (51) |  |
| **Clinical presentation** |  |  |  |
| Dyspnoea | 206 (96.7) | 152 (96.8) | 0.877 |
| Orthopnoea | 93 (43.7) | 76 (48.4) | 0.224 |
| Weight gain | 60 (28.2) | 42 (26.8) | 0.719 |
| Jugular venous distension * | 93 (43.7) | 73 (46.5) | 0.981 |
| Fatigue | 19 (8.9) | 14 (8.9) | 1.000 |
| Cold extremities | 6 (2.8) | 4 (2.5) | 0.873 |
| Pulmonary rales | 156 (73.2) | 112 (71.3) | 0.648 |
| Peripheral oedema | 154 (72.3) | 112 (71.3) | 0.877 |
| Chest Pain | 43 (20.2) | 39 (24.8) | 0.300 |
| Rhythm |  |  | 0.198 |
| -SR | 114/210 (54.3) | 96/156 (61.5) |  |
| -AF | 84/210 (40) | 47/156 (30.1) |  |
| -Other | 12/210 (5.7) | 13/156 (8.3) |  |
| BMI, kg/m^2^ | 28.2±6.2 | 27.5±5.9 | 0.334 |
| SBP, mmHg | 145.3±31.4 | 137.9±29.7 | *0.023* |
| DBP, mmHg | 80.7±19.2 | 79.1±18.7 | 0.438 |
| HR, bpm | 96.8±28.9 | 92.9±26.8 | 0.196 |
| O_2_ support | 54 (25.4) | 44 (28) | 0.282 |
| Chest X-ray congestion | 166 (77.9) | 118 (75.2) | 0.551 |
| **Echo** |  |  |  |
| LVEF | 43.5±15.1 | 43.8±17.3 | 0.889 |
| Type of HF*  HFrEF  HFmrEF  HFpEF | 61 (38.4%)  40 (25.2%)  58 (36.5%) | 55 (42.3%)  15 (11.5%)  60 (46.2%) | 0.012 |
| **Laboratory values** |  |  |  |
| s-Cr, umol/l | 109 (87-142) | 116 (93-164) | 0.085 |
| eGFR, ml/min | 53±29 | 51±30 | 0.366 |
| Urea, mmol/l | 9.2 (6.7-12.9) | 9.7 (6.9-16.3) | 0.073 |
| K+, mmol/l | 4.63±0.96 | 4.66±1.16 | 0.799 |
| Sodium, mmol/l | 139 (136-141) | 139 (134.8-141) | 0.233 |
| NT-proBNP, pmol/l | 633 [328-1488] | 718 [389-1402] | 0.363 |
| Log NT-proBNP | 2.82±0.45 | 2.86±0.45 | 0.471 |
| **Admission Medications** |  |  |  |
| Beta-blocker | 132 (62) | 107 (68.2) | 0.209 |
| ACE-I | 63 (29.6) | 52 (33.1) | 0.420 |
| ARB | 49 (23) | 46 (29.3) | 0.157 |
| Nitrates | 41 (19.2) | 44 (28) | *0.042* |
| MRA | 27 (12.7) | 25 (15.9) | 0.336 |
| HCT | 25 (11.7) | 20 (12.7) | 0.749 |
| Loop diuretics | 96 (45.1) | 94 (59.9) | *0.003* |
| Daily loop diuretic dose, mg/day | 82±88.5 | 113.4±99.9 | *0.024* |
| **Hospitalization therapy** |  |  |  |
| Beta-blocker | 173 (81.2) | 126 (80.3) | 0.816 |
| ACE-I | 113 (53.1) | 75 (47.8) | 0.315 |
| ARB | 46 (21.6) | 42 (26.8) | 0.250 |
| MRA | 55 (25.8) | 42 (26.8) | 0.841 |
| HCT | 28 (13.1) | 16 (10.2) | 0.368 |
| Nitrates | 133 (62.4) | 99 (63.1) | 0.904 |
| Inotropes | 10 (4.7) | 10 (6.4) | 0.481 |
| Mean daily loop diuretic dose, mg/day | 110 [70-161] | 130 [88-192] | *0.007* |

Data are presented as mean ± SD, median (interquartile range) or number (percentage) of patients.

Abbreviations as in Table S1. *The difference between percentages could be affected by missing data.

# Hb<7.5 mmol/l in women or <8.1 mmol/l in men.

## **Table S3. Characteristics of patients with and without one-year CV mortality.**

| **Variable** | **No CV mortality**  **N=308** | **CV mortality**  **N=62** | **p Value** |
| --- | --- | --- | --- |
| **Demographics** |  |  |  |
| Age, years | 76.2±12.3 | 77.9±10.1 | 0.301 |
| Male sex | 162 (52.6) | 33 (53.2) | 0.928 |
| **Medical History** |  |  |  |
| Obesity | 94 (30.5) | 16 (25.8) | 0.459 |
| Hypertension | 168 (54.5) | 31 (50) | 0.664 |
| Dyslipidemia | 63 (20.5) | 12 (19.4) | 0.937 |
| Diabetes mellitus | 102 (33.1) | 24 (38.7) | 0.358 |
| Smoking | 88 (28.6) | 9 (14.5) | *0.024* |
| CKD | 48 (15.6) | 16 (25.8) | *0.046* |
| COPD | 56 (18.2) | 12 (19.4) | 0.837 |
| SID | 26 (8.4) | 2 (3.2) | 0.164 |
| Cancer | 60 (19.5) | 7 (11.3) | 0.136 |
| Anaemia | 36 (11.7) | 7 (11.3) | 0.962 |
| CAD | 123 (39.9) | 34 (54.8) | *0.032* |
| Previous PCI | 48 (15.6) | 10 (16.1) | 0.922 |
| Previous CABG | 49 (15.9) | 21 (33.9) | *0.001* |
| Primary CMP | 6 (1.9) | 2 (3.2) | 0.514 |
| PAD | 61 (19.8) | 13 (21) | 0.863 |
| Arrhythmia | 118 (38.3) | 25 (40.3) | 0.767 |
| Atrial fibrillation | 104 (33.8) | 24 (38.7) | 0.455 |
| HVD | 78 (25.31) | 21 (33.9) | 0.170 |
| Pulmonary Hypertension | 15 (4.9) | 7 (11.3) | *0.047* |
| Stroke/TIA | 56 (18.2) | 12 (19.4) | 0.828 |
| **Triggers events for AHF** |  |  | 0.065 |
| -ACS | 38 (12.3) | 6 (9.7) |  |
| -Acute Kidney Disease | 2 (0.6) | 2 (3.2) |  |
| -Anaemia^#^ | 6 (1.9) | 1 (1.6) |  |
| -Arrhythmias | 76 (24.7) | 8 (12.9) |  |
| -CS | 4 (1.3) | 0 |  |
| -Fluid Overloading | 79 (25.6) | 22 (35.5) |  |
| -Hypertension | 26 (8.4) | 3 (4.8) |  |
| -Mechanical cause | 23 (7.5) | 12 (19.4) |  |
| -Non adherence to medication or diet | 3 (1) | 1 (1.6) |  |
| -PE | 1 (0.3) | 1 (1.6) |  |
| -Primary CMP | 6 (1.9) | 1 (1.6) |  |
| -Respiratory infection | 26 (8.4) | 3 (4.8) |  |
| -Toxic damage | 3 (1) | 0 |  |
| -Others | 15 (4.9) | 2 (3.2) |  |
| Setting |  |  | 0.801 |
| -In Hospital | 12 (3.9) | 2 (3.2) |  |
| -Out Hospital | 296 (96.1) | 60 (96.8) |  |
| Chronic vs De Novo |  |  | *0.002* |
| -CHF | 119 (38.6) | 37 (59.7) |  |
| -De novo HF | 189 (61.4) | 25 (40.3) |  |
| **Clinical presentation** |  |  |  |
| Dyspnoea | 298 (96.8) | 60 (96.8) | 0.571 |
| Orthopnoea | 140 (45.5) | 29 (46.8) | 0.411 |
| Weight gain | 82 (26.6) | 20 (32.3) | 0.269 |
| Jugular venous distension * | 139 (45.1) | 27 (43.5) | 0.153 |
| Fatigue | 28 (9.1) | 5 (8.1) | 0.790 |
| Cold extremities | 8 (2.6) | 2 (3.2) | 0.778 |
| Pulmonary rales | 222 (72.1) | 46 (74.2) | 0.820 |
| Peripheral oedema | 219 (71.1) | 47 (75.8) | 0.401 |
| Chest Pain | 70 (22.7) | 12 (19.4) | 0.558 |
| Rhythm |  |  | 0.089 |
| -SR | 170/304 (55.9) | 40 (64.5) |  |
| -AF | 116/304 (38.2) | 15 (24.2) |  |
| -Other | 18/304 (5.9) | 7 (11.3) |  |
| BMI, kg/m^2^ | 28±6.1 | 27.3±5.9 | 0.411 |
| SBP, mmHg | 144.8±31.2 | 129.3±25.6 | *0.000* |
| DBP, mmHg | 81.2±19.3 | 74.2±16.4 | *0.008* |
| HR, bpm | 96.9±28.6 | 86.6±23.5 | *0.009* |
| O2 support | 81 (26.3) | 17 (27.4) | 0.479 |
| Chest X-ray congestion | 236 (76.6) | 48 (77.4) | 0.757 |
| **Echo** |  |  |  |
| LVEF | 44.3±15.5 | 40.8±18.2 | 0.141 |
| Type of HF*  HFrEF  HFmrEF  HFpEF | 88 (37.6%)  49 (20.9%)  97 (41.5%) | 28 (50.9%)  6 (10.9.7%)  21 (38.2%) | 0.109 |
| **Laboratory values** |  |  |  |
| s-Cr, umol/l | 109 (87-141) | 133 (102-188) | *0.001* |
| eGFR, ml/min | 54±30 | 41±20 | *0.001* |
| Urea, mmol/l | 8.9 (6.5-12.9) | 13.7 (9.6-21) | *0.000* |
| K+, mmol/l | 4.65±1.1 | 4.61±0.86 | 0.805 |
| Sodium, mmol/l | 139 (136-141) | 136 (133.8-140) | *0.002* |
| Nt-proBNP, pmol/l | 1113±2280 | 1750±1751 | 0.065 |
| Log NT-proBNP | 3.01 (2.80-3.26) | 3.13 (2.91-3.30) | 0.000 |
| **Admission Medications (%)** |  |  |  |
| Beta-blocker | 191 (62) | 48 (77.4) | *0.011* |
| ACE-I | 94 (30.5) | 21 (33.9) | 0.545 |
| ARB | 79 (25.6) | 16 (25.8) | 0.935 |
| MRA | 38 (12.3) | 14 (22.6) | *0.025* |
| HCT | 37 (12) | 8 (12.9) | 0.824 |
| Nitrates | 68 (22.1) | 17 (27.4) | 0.345 |
| Loop diuretics | 145 (47.1) | 45 (72.6) | *0.000* |
| Daily loop diuretic dose, mg/day | 93.2±95.3 | 111.8±95.4 | 0.255 |
| **Hospitalization therapy** |  |  |  |
| Beta-blocker | 250 (81.2) | 49 (79) | 0.697 |
| ACE-I | 162 (52.6) | 26 (41.9) | 0.125 |
| ARB | 75 (24.4) | 13 (21) | 0.568 |
| Nitrates | 194 (63) | 38 (61.3) | 0.801 |
| MRA | 73 (23.7) | 24 (38.7) | *0.014* |
| HCT | 36 (11.7) | 8 (12.9) | 0.787 |
| Inotropes | 12 (3.9) | 8 (12.9) | *0.004* |
| Mean daily loop diuretic dose, mg/day | 130±81.9 | 182.5±99.1 | *0.000* |

Data are presented as mean ± SD, median (interquartile range) or number (percentage) of patients.

Abbreviations as in Table S1. *The difference between percentages could be affected by missing data.

# Hb<7.5 mmol/l in women or <8.1 mmol/l in men.
